# Draft genome sequence data of heavy metal-resistant *Morganella morganii* WA01/MUTU, a silver nanoparticle (AgNP) synthesising bacterium

**DOI:** 10.1016/j.dib.2023.109873

**Published:** 2023-11-30

**Authors:** Montri Yasawong, Prapimpun Wongchitrat, Chartchalerm Isarankura-Na-Ayudhya, Patcharee Isarankura-Na-Ayudhya, Piyada Na Nakorn

**Affiliations:** a Program on Environmental Toxicology, Chulabhorn Graduate Institute, Chulabhorn Royal Academy, Bangkok 10210, Thailand; b Center of Excellence on Environmental Health and Toxicology (EHT), OPS, MHESI, Bangkok 10400, Thailand; c Center for Research Innovation and Biomedical Informatics, Faculty of Medical Technology, Mahidol University, Nakhon Pathom 73170, Thailand; d Department of Clinical Microbiology and Applied Technology, Faculty of Medical Technology, Mahidol University, Nakhon Pathom 73170, Thailand; e Department of Medical Technology, Faculty of Allied Health Sciences, Thammasat University, Pathumthani 12120, Thailand

**Keywords:** Heavy metal resistance, AgNP synthesis, *Morganella morganii*, Bacterial genome

## Abstract

*Morganella morganii* WA01/MUTU is a heavy metal tolerant strain capable of producing silver nanoparticles (AgNPs) from AgNO_3_. Here we present the draft genome sequence of *M. morganii* WA01/MUTU isolated from a water sample collected in Nakhon Pathom province, Thailand. The draft genome was sequenced on the Illumina NextSeq 550 sequencer. The genome consisted of 34 contigs with a total size of 3,991,804 bp, an N50 value of 364,423 bp and a GC content of 50.93%. The digital DNA-DNA hybridisation (dDDH) between WA01/MUTU and *Morganella morganii* (NBRC 3848) was 83.9%, identifying the strain as *Morganella morganii*. The data presented here can be used in comparative genomics to identify gene clusters involved in AgNP biosynthesis and secondary metabolite production. The draft genome sequence data was deposited at NCBI under Bioproject accession number PRJNA493966.

Specifications Table

**Table:** 

Subject	Biological sciences
Specific subject area	Omics: Genomics
Data format	Raw and analysed
Type of data	Table, figure
Data collection	DNA was extracted using the Zymo Quick-DNA HMW MagBead Kit and sequenced with an Illumina NextSeq 550. Adapter trimming and quality filtering were carried out using AfterQC v0.9.6. Unicycler v0.5.0 was utilised for genome assembly while QUAST v5.0.2 was employed for assembly metrics determinations. Genome quality was evaluated using CheckM v1.1.2. Digital DNA-DNA hybridisations and a phylogenomic tree were analysed on the Type (Strain) Genome Server. The genome map was produced using Proksee. The NCBI Prokaryotic Genome Annotation Pipeline was utilised for genome annotation, whereas antiSMASH v7.0.1 was employed for the identification of secondary metabolite biosynthesis gene clusters.
Data source location	*Morganella moganii* WA01/MUTU was isolated from a water sample collected in Nakhon Pathom province, Thailand (N 13º 43′ 55.518″, E 100º 11′ 15.373″).
Data accessibility	The sequencing data were deposited in the National Center for Biotechnology Information (NCBI) Genbank database under accession number JAVJNK000000000. The deposited draft genome sequencing data are available at https://www.ncbi.nlm.nih.gov/nuccore/JAVJNK000000000.

## 1 Value of the Data

• The draft genome sequence of M. morganii WA01/MUTU may be useful for comparative genomic studies with other Morganella species.
• Elucidation of the genome sequence of M. morganii WA01/MUTU may help to characterise the genes involved in AgNP biosynthesis.
• Elucidation of the genome sequence of M. morganii WA01/MUTU may help in the discovery of novel gene clusters encoding bioactive compounds.

## 2 Data Description

*Morganella morganii* is Gram-negative, facultatively anaerobic [1] and motile with peritrichous flagella [2]. Cells of *M. morganii* are short, straight rods and do not produce pigment or capsules [3]. Growth of *M. morganii* occurs between 4 °C and 45 °C on nutrient agar and colonies are smooth, transparent with an entire edge [3]. Some species of the genus *Morganella* are capable of synthesising metal nanoparticles in the aqueous phase [4,5]. Furthermore, there is a strong correlation between the ability of different bacterial strains to synthesise metal nanoparticles and the heavy metal resistance mechanism [6]. These biosynthesised NPs have potential use in several medical applications, including antibacterial and drug delivery [7,8]. The minimum inhibitory concentration (MIC) of strain WA01/MUTU against CdCl_2_ was 3.2 mM. The silver nanoparticles (AgNPs) produced by WA01/MUTU have a spherical shape with a diameter of approximately 15–20 nm. Therefore, we analysed the whole genome sequence of *M. morganii* WA01/MUTU to obtain insight into possible genes that involved in NP synthesis and to extend our knowledge of secondary metabolite production.

**Fig. 1:**
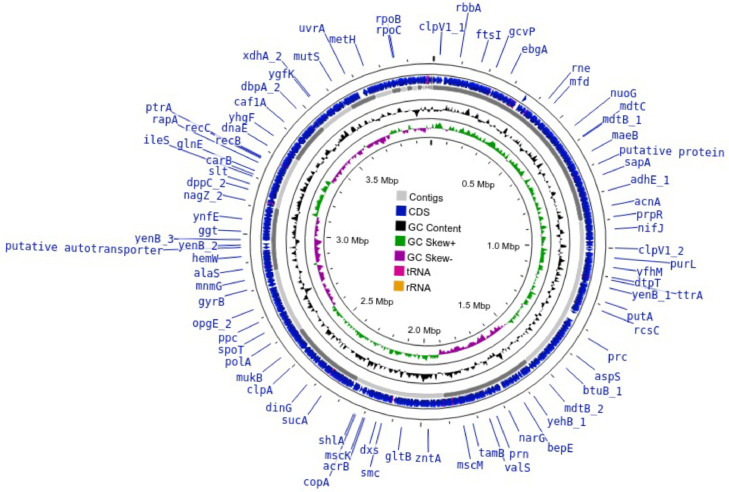
Genome map of *M. morganii* WA01/MUTU. The map consists of blue arrows indicating CDSs, grey arrows indicating contigs, green peaks representing GC skew+, purple peaks representing GC skew-, and black peaks representing GC content. (For interpretation of the references to color in this figure legend, the reader is referred to the web version of this article.)

**Fig. 2:**
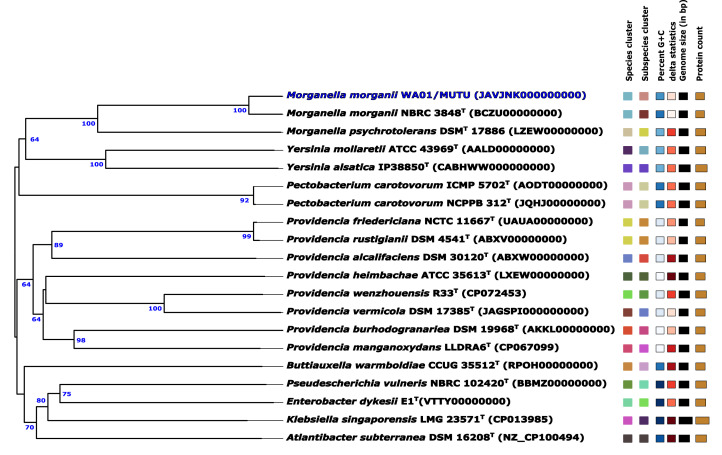
Phylogenomic tree based on the whole-genome sequence data result of WA01/MUTU and closely related type strains reconstructed on the TYGS. Branch numbers are Genome Blast Distance Phylogeny (GBDP) pseudo-bootstrap support values > 60% from 100 replicates, with an average branch support of 78.6%.

The draft genome sequence of strain WA01/MUTU (accession number JAVJNK000000000) is available at the National Center for Biotechnology Information (NCBI) under the BioProject number PRJNA493966 and the BioSample number SAMN37308709 (Table 1). Raw reads were deposited in the NCBI Sequence Read Archive (SRA) database (SRR25935570) (Table 1). Here we present data on the draft genome sequence of *M. morganii* WA01/MUTU (Fig. 1), including its potential secondary metabolite biosynthesis. The draft genome of strain WA01/MUTU consists of 34 contigs with a total length of 3,991,804 base pairs. The N50 was 364,423 bp and the GC content was 50.93% (Table 1). The CheckM reported 100% completeness with an estimated contamination of less than 1% for the WA01/MUTU draft genome sequence. The bacterial strain WA01/MUTU was affiliated to *Morganella morganii* (NBRC 3848) with a digital DNA-DNA hybridisation (dDDH) value of 83.9% [9]. The phylogenomic tree of the strain WA01/MUTU and closely related strains is shown in Fig. 2.

**Table 1:** Genomic features and assembly statistics for *M. morganii* WA01/MUTU.

Attribute	*M. morganii* WA01/MUTU
Genome size (bp)	3,991,804
Number of contigs	34
Genome coverage	199×
GC content (%)	50.93
Largest contig (bp)	892,956
N50 (bp)	364,423
N75 (bp)	256,812
L50	4
L75	7
Total gene	3739
Total CDS	3621
tRNA	69
rRNA	4
ncRNA	4
BioProject	PRJNA493966
BioSample	SAMN37308709
Accession Number	JAVJNK000000000.1
Sequence Read Archive (SRA)	SRR25935570

A cluster of nitrate reductase genes, which play an important role in AgNP biosynthesis [10], was observed in the draft genome sequence of strain WA01/MUTU. The genome mining study revealed three potential secondary metabolic gene clusters, including betalactone, thiopeptide and arylpolyene, were observed within three regions with varying percentages of similarity. The highest cluster similarity was 84% for arylpolyene (APE), corresponding to bacterial pigment production [11]. We believe that the draft genome sequence will facilitate the characterisation of genes involved in the biosynthesis of AgNPs and secondary metabolites in *M. morganii* WA01/MUTU.

## 3 Experimental Design, Material and Methods

### 3.1 Bacterial isolation

Strain WA01/MUTU was isolated from a water sample collected in Nakhon Pathom, Thailand (N 13º 43′ 55.518″, E 100º 11′ 15.373″). For screening experiment, the water sample was spread on Luria-Betani (LB) agar containing 0.4 mM CdCl_2_ and incubated at 37 °C for 18 h. The 0.4 mM cadmium (Cd) resistant strains were isolated by cross-streaking on LB agar plates and incubated at 37 °C for 18 h. For the heavy metal tolerance test, the resistant strains were tested against 2-fold concentrations of CdCl_2_ (ranging from 0.1–3.2 mM) on LB agar. A single colony of strain WA01/MUTU at the minimum inhibitory concentration (MIC) of CdCl_2_ was grown in LB broth overnight at 37 °C with shaking at 250 rpm. The ability of strain WA01/MUTU to synthesise extracellular silver nanoparticles (AgNPs) was observed by treatment with 5 mM AgNO_3_ and incubation for 20 h at 37 °C with shaking at 180 rpm. The characteristic of AgNPs produced by strain WA01/MUTU was determined by ultraviolet-visible (UV-Vis) spectroscopy (Shimadzu, Japan), and surface plasmon resonance (SRP) at 350-530 nm was used to determine the characteristic peak of AgNPs. In addition, the morphology and size of AgNPs were determined by transmission electron microscopy (JOEL, Japan).

### 3.2 Genomic DNA (gDNA) preparation

High quality gDNA from the strain WA01/MUTU was obtained from overnight cultures using the Quick-DNA HMW MagBead Kit (Zymo, USA). The concentration of gDNA was determined using NanoDrop spectrophotometry (Thermo Scientific, USA).

### 3.3 Whole genome sequencing and assembly

Sequencing libraries were prepared from 1 ng of DNA using the Nextera XT DNA library preparation kit (Illumina, San Diego, CA, USA). Raw sequencing reads were acquired on a NextSeq 550 sequencer using the NextSeq 500/550 high output kit v2.5 (300 cycles, 2×150-bp reads) (Illumina). AfterQC v0.9.6 with default parameters [12] was used for quality checks, adapter trimming and quality filtering. *De novo* genome assembly was performed using the raw reads and Unicycler v0.5.0 with default parameters [13]. Genome assembly metrics were determined using QUAST v5.0.2 with default parameters [14]. The genome map of strain WA01/MUTU was constructed using Proksee [15].

### 3.4 Taxonomic identification of the strain

Genome quality assessment was performed using CheckM v1.1.2 with default parameters [16]. Digital DNA-DNA hybridisation (dDDH) and a phylogenomic tree based on the whole genome sequence of WA01/MUTU and closely related strains were performed using the Type (Strain) Genome Server (TYGS) [9].

### 3.5 Genome annotation and sequence analysis

Genome annotation was performed using the NCBI Prokaryotic Genome Annotation Pipeline (PGAP) with default parameters [17], and genome mining for potential secondary metabolites was performed using antiSMASH v7.0.1 with default parameters [18].

## Limitations

The use of next-generation sequencing techniques generates vast quantities of data. However, de novo genome assemblies resulting from this data often display significant deficiencies in completeness. The current assemblies possess shortcomings that render them vulnerable to annotation errors, particularly regarding the imprecise estimation of genes that could possibly exist in the draft genome of WA01/MUTU.

## Ethics Statement

This work does not involve human subjects, animal experiments, or any data collected from social media platforms.

## CRediT authorship contribution statement

**Montri Yasawong:** Methodology, Data curation, Writing – original draft, Writing – review & editing. **Prapimpun Wongchitrat:** Conceptualization, Data curation. **Chartchalerm Isarankura-Na-Ayudhya:** Data curation. **Patcharee Isarankura-Na-Ayudhya:** Supervision, Writing – original draft, Writing – review & editing. **Piyada Na Nakorn:** Methodology, Supervision, Writing – original draft, Writing – review & editing.

## Data Availability

Morganella morganii strain WA01/MUTU, whole genome shotgun sequencing project (Original data) (NCBI)
